# Comparison of velopharyngeal morphology of two palatoplasty techniques in patients with hard and soft cleft palate

**DOI:** 10.3389/fsurg.2022.1080955

**Published:** 2023-01-06

**Authors:** Xiaofen Fan, Weilong Liu, Jiancun Nie, Xiaoxuan Chen, Yingchun Dong, Yong Lu

**Affiliations:** ^1^ Department of Oral and Maxillofacial Surgery, Nanjing Stomatological Hospital, Medical School of Nanjing University, Nanjing, China; ^2^ Department of Oral Anesthesiology, Nanjing Stomatological Hospital, Medical School of Nanjing University, Nanjing, China

**Keywords:** cleft palate, velopharyngeal function, speech outcome, furlow, sommerlad

## Abstract

**Purpose:**

The study aims to compare the velopharyngeal morphology of hard and soft cleft palate (HSCP) patients after Furlow and Sommerlad palatoplasty.

**Patients and methods:**

A total of 51 patients (20 cases in Furlow palatoplasty group, 16 cases in Sommerlad palatoplasty group and 15 normal children in the control group) were included in our study. Velopharyngeal function and speech outcomes of patients with HSCP who had either Furlow palatoplasty or Sommerlad palatoplasty for cleft palate repair were evaluated by perceptual speech assessment (PSA), lateral cephalometric radiographs and nasopharyngoscopy. To assess velopharyngeal morphology of patients treated with two techqiques, we analyzed measurements such as velar length, pharyngeal depth, and the Adequate ratio (the ratio of velar length to pharyngeal depth). Furthermore, skeletal landmarks including cranial base, cervical vertebrae, posterior nasal spine which were defined as the pharyngeal triangle were measured. Finally, the position of the point U relative to the pharyngeal triangle were compared.

**Results:**

Velopharyngeal closure (VPC) rate in Furlow palatoplasty group accounted for 90%, while that in Sommerlad palatoplasty group was 81.3%. PSA of the former group was significantly better than that of the latter group (*P* < 0.05). Velar length, pharyngeal depth and the Adequate ratio (1.37 ± 0.14 vs. 1.41 ± 0.15) were comparable between the Furlow group and control group (*P* > 0.05), while Sommerlad group had a shorter velar length, deeper pharyngeal depth and a smaller Adequate ratio (1.20 ± 0.18) compared to the above two groups (*P* < 0.05). Furhermore, the point U of Sommerlad group in the pharyngeal triangle was higher than that of the other two groups.

**Conclusions:**

In the treatment modality of patients with HSCP, both Furlow palatoplasty and Sommerlad palatoplasty seem to be effective. Furlow palatoplasty appears to have velopharyngeal morphology similar to normal control group., while Sommerlad group shows a shorter velar length, deeper pharyngeal depth and a smaller Adequate ratio

## Introduction

Cleft palate is the most common congenital developmental deformity of the maxillofacial region, the incidence of it lies on top of congenital birth defect diseases (1–3). It exerts a serious socio-economic and psychosocial burden on patients and their families (4–6). Currently, surgical intervention has still emerged as the first treatment choice. Although various techniques have been described for cleft palate repair, however, there is no consensus on the ideal palatal repair technique applicable to all cleft palate types (7–10). The primary goal of cleft palate repair is the achievement of optimal speech outcomes (11, 12). To accomplish this goal, anatomical separation between the oral and nasal cavities and restoration of the muscular sling in the soft palate is pivotal (13). Furlow palatoplasty proposed by Furlow in 1986, and Sommerlad palatoplasty described by Sommerlad in 2002 were currently two popular methods to achieve VPC and yield intelligible speech by means of anatomical reconstruction of the levator palatine muscle (14, 15). Whereas the way restoring abnormally attached muscles was different. The former was by means of dissection and reposition of the levator veli palatini, while the latter was achieved with the help of the double-opposing Z-plasty. Previously, several studies had evaluated the clinical outcomes of these two surgical procedures (16–20). Khosla et al. reported Furlow Z-plasty yielded excellent speech results for primary cleft palate repair, and the rate of VPC reached 84% (16). According to Wang et al., 80% patients with Sommerlad palatoplasty had no evidence of velopharyngeal insufficiency (18). In addition, Li et al. compared the incidence of postoperative fistula formation of the above two techniques and found that the fistula rate of Furlow palatoplasty is lower than that of Sommerlad palatoplasty (19). However, few research have been conducted to compare the velopharyngeal morphology between Furlow and Sommerlad techniques. If any, they were assessed only using subjective evaluation methods (18). Thus, the current study aims to compare velopharyngeal morphology and speech outcomes using subjective and objective parameters after cleft palate repair with Furlow or Sommerlad palatoplasty, to provide clinical evidence for a better choice of surgical methods to reconstruct a functional velopharyngeal unit and restore optimal speech in patients with HSCP.

## Materials and methods

### Subjects

Medical records of patients with HSCP who underwent primary cleft palate repair in Nanjing Stomatological Hospital, Medical School of Nanjing University between 2012 and 2018 were reviewed retrospectively. All patients enrolled in the study met the following inclusion criteria: patients with HSCP; non-syndromic patients; and primary cleft palate repair using Furlow or Sommerlad palatoplasty. Patients were excluded for syndromic diseases, and for a history of delayed language development and dysacusia.

This study was approved by the Ethics Committee, Nanjing Stomatological Hospital, Medical School of Nanjing University.

Patients with HSCP underwent surgical repair by Furlow or Sommerlad palatoplasty. Their ages performing surgical procedures ranged from 9 months to 12 months. The mean age at follow-up was similar between groups. Patients were randomly assigned to Furlow palatoplasty group (*n* = 20, 9 males and 11females) and Sommerlad palatoplasty group (*n* = 16, 7 males and 9 females). Surgical procedures were performed by two senior surgeons, respectively (14, 15). Fifteen healthy children (good articulation, no hearing handicap, no cleft lip or cleft palate, no congenital maxillofacial developmental malformations, no orthodontic treatment history) were selected as the control group. Details were provided in Table 1.

**Table 1:** Age and gender distribution of the subjects.

		Surgical age (month)			
	*n*	Mean	Range	Male	Female
T1 group	20	10.5 months	9–12 months	9	11
T2 group	16	11 months	9–12 months	7	9

T1 group, furlow palatoplasty; T2 group, sommerlad palatoplasty.

### Perceptual speech assessment

Speech evaluation was evaluated by two experienced speech pathologists using a speech articulation test table (21). Patients were asked to read a standard list of 63 phrases that cover all phonemes and most common phonetic combinations in Mandarin Chinese, and 20 short sentences containing voiced and unvoiced pressure consonants, no-pressure consonants, and a mixture of nasal consonants. The next part of the test was a casual conversation between the participants and the speech pathologist, in which speech intelligibility, hypernasality and nasal emission were measured. These parameters were evaluated following the scoring guidelines and definitions of the CAPS-A protocol (22). Intelligibility and hypernasality were evaluated using a five-point scale, whereas for the evaluation of nasal emission a three-point scale was used. Interrater and intrarater reliabilities were investigated using weighted kappa statistics to assess observer variability. Each speech language pathologist rated speech after listening to 20 recorded speech samples and the ratings were compared to determine agreement between the two raters. The ratings were made twice with an interval of one week by each rater and two ratings by the same rater were compared.

### Nasoendoscopy

Nasoendoscopy was performed for patients diagnosed with hypernasality or nasal emission that were cooperative, usually over the age of 4 years. Nasoendoscopy was used to observe the motility of the velum and posterior pharyngeal wall and lateral pharyngeal wall of patients with VPI. The results were diagnosed as VPC and VPI.

### Lateral cephalometric assessments

**Figure 1:**
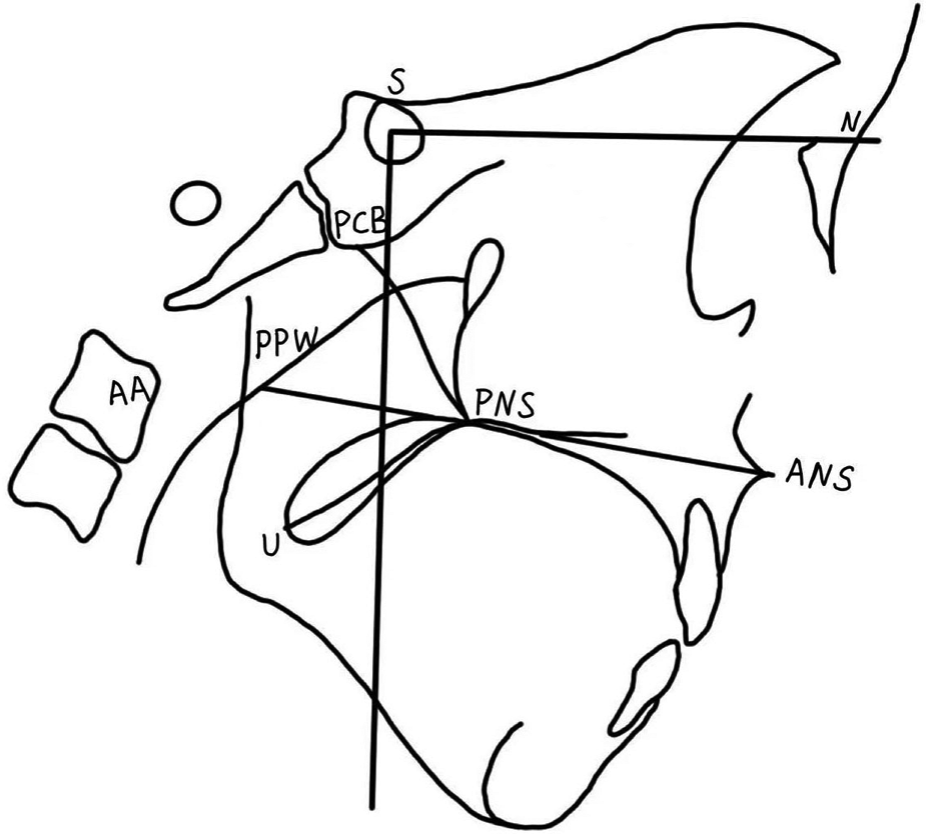
Cephalometric landmarks and velopharyngeal measures. ANS, anterior nasal spine; PNS, posterior nasal spine; PPW, posterior pharyngeal wall; N, nasion; S, center of the sella turcica; PCB, pterygoid cranial base; AA, atlas; U, uvula, tip of the uvula when at rest.

Set points (Figure 1): ANS, anterior nasal spine; PNS, posterior nasal spine; PPW, posterior pharyngeal wall; N, nasion; S, center of the sella turcica; PCB, pterygoid cranial base; AA, atlas; U, uvula, tip of the uvula when at rest.

**Figure 2:**
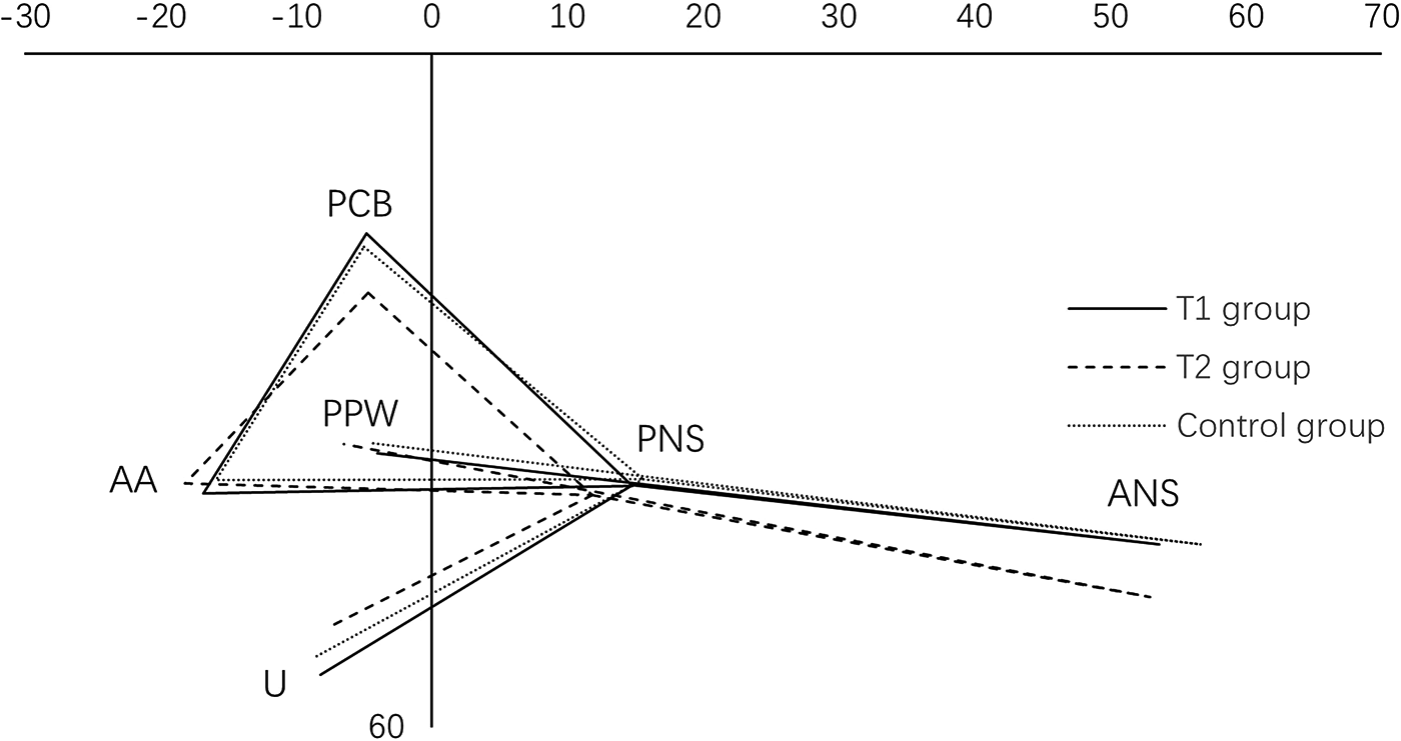
Velopharyngeal morphology of three groups. ANS, anterior nasal spine; PNS, posterior nasal spine; PPW, posterior pharyngeal wall; N, nasion; S, center of the sella turcica; PCB, pterygoid cranial base; AA, atlas; U, uvula, tip of the uvula when at rest.

Measurement methods: To eliminate the interference of age and sex among the 3 groups, all craniopharyngeal dimensions were standardized according to the anterior cranial base length (S–N) with a given value of 100 (S–N revision). Measurements derived from tracing of lateral cephalograms by drawing the sella nasion (S–N) plane as the x-axis and projecting a perpendicular line to this plane through the point S as the y-axis. The length of the velar PNS-U, the depth of the nasopharynx PNS-PPW and the length of the hard palate ANS-PNX (Figure 1) were measured, and the adequate ratio (velar length to pharyngeal depth ratio) was calculated. For the three groups, ANS, PNS, PCB, AA, U, PPW were marked in the coordinates according to their (*x*, *y*) values. To facilitate the comparison of velopharyngeal morphology among the 3 groups, the triangle connecting PNS, PCB, and AA was defined as the pharyngeal triangle (Figure 2). Each measurement was performed twice by the first author, and the mean value was recorded.

### Statistical analysis

The Wilcoxon rank sum test was used to compare the speech outcomes. The results of cephalometric measurements of the three groups were subscribed to one-way ANOVA using IBM SPSS (version 26.0). Differences with *P* < 0.05 were considered statistically significant.

## Results

### Speech outcomes

The kappa values for interrater reliability were 0.67, and for intrarater reliability were 0.72 and 0.68, indicating substantial agreement. The results of PSA were shown in Table 3. The speech intelligibility in Furlow group was significantly better than that in Sommerlad palatoplasty (*P* = 0.023). Furlow palatoplasty's hypernasality improved better than Sommerlad palatoplasty (*P* = 0.009). Postoperative nasal emissions differed significantly between the two groups (*P* = 0.016) (Table 2).

**Table 2:** Comparative analysis of speech intelligibility, hypernasality and nasal emission in furlow and sommerlad palatoplasty groups.

	T1 group (*n* = 20)	T2 group (*n* = 16)	*P*
**Speech intelligibility**			
0: normal	13	4	0.023
1: different, not enough to cause comment	4	6	
2: different enough to cause comment, but intelligible t	1	3	
3: just intelligible to strangers	2	2	
4: impossible to understand	0	1	
**Hypernasality**			
0: absent	14	4	0.009
1: borderline	3	6	
2: mild	2	2	
3: moderate	1	3	
4: severe	0	1	
**Nasal emission**			
0: absent	14	5	0.016
1: occasionally heard	5	7	
2: Nasal emission	1	4	

T1 group, furlow palatoplasty; T2 group, sommerlad palatoplasty.

**Table 3:** Comparison of cephalometric landmarks among three groups.

Item	T1 Group	T2 Group	Control
ANS (*x*)	53.6 ± 2.7	52.9 ± 4.2	57.2 ± 3.7
(*y*)	43.6 ± 4.3	48.4 ± 4.3	43.7 ± 4.5
PNS (*x*)	14.8 ± 3.1	11.8 ± 4.3	15.6 ± 3.9
(*y*)	38.5 ± 3.6	39.3 ± 4.3	37.9 ± 3.6
PCB (*x*)	−4.6 ± 0.4	−4.8 ± 0.8	−5.1 ± 0.8
(*y*)	16.0 ± 2.1	21.3 ± 3.2	17.2 ± 2.6
PPW (*x*)	−4.0 ± 2.6	−6.5 ± 3.4	−4.3 ± 4.0
(*y*)	35.9 ± 3.6	34.8 ± 7.9	34.7 ± 5.0
AA (*x*)	−16.8 ± 4.1	−18.2 ± 4.8	−15.9 ± 5.9
(*y*)	39.5 ± 5.0	38.3 ± 4.6	38.0 ± 5.7
U (*x*)	−8.2 ± 3.6	−7.6 ± 3.2	−8.5 ± 4.5
(*y*)	55.3 ± 5.5	51.1 ± 6.1	53.7 ± 5.8
Velar length/mm	28.2 ± 3.2	25.0 ± 3.8	29.2 ± 3.6
Pharyngeal depth/mm	20.4 ± 2.5	21.1 ± 3.6	20.7 ± 3.3
Adequate ratio	1.37 ± 0.14	1.20 ± 0.18	1.41 ± 0.15

T1 group, furlow palatoplasty; T2 group, sommerlad palatoplasty.

ANS, anterior nasal spine; PNS, posterior nasal spine; PPW, posterior pharyngeal wall; N, nasion; S, center of the sella turcica; PCB, pterygoid cranial base; AA, atlas; U, uvula, tip of the uvula when at rest.

### Nasoendoscopy

3 patients of T1 group and 4 patients of T2 group were recommended to perform nasoendoscopy. There were 2(20) and 3(16) patients who were diagnosed with having VPI, respectively. Eventually, the rate of VPC in T1 group (90%) was higher than that in T2 group (81.3%).

### Lateral cephalogram evaluation

The interclass correlation coefficient results for the test-retest reliability ranged between 0.72 and 0.83 (*P* < 0.05), suggesting dependable reliability and reproducibility of the adopted measuring strategy. The results of cephalometric measurements for the velopharyngeal structure of the three groups were presented in Table 3. There was no significant difference in the coordinates of points PNS, PCB, and AA on either axis among the 3 groups (*P* > 0.05). The velar length and Adequate ratio of T1 group was similar to that of the normal group (*P* > 0.05). The velopharyngeal structure was also similar to that of the control group. The velopharyngeal depths of the three groups were similar (*P* > 0.05). Velar length and adequate ratio were significantly smaller in T2 group than in the other two groups (*P* < 0.05). Adequate ratio averaged more than 1.3 in T1 group and control group. The point U of T2 group in the pharyngeal triangle was above that of the other two groups. The coordinate of point ANS on the x-axis in the T1 and T2 group was inferior to that in the control group (*P* < 0.05).

## Discussion

The main objective of cleft palate repair is to reconstruct proper velopharyngeal function for normal speech intelligibility (19), which requires restoration of an anatomically well-integrated dynamic and functional soft palate. Furlow and Sommerlad methods are two well-established methods to optimize velopharyngeal function by restoring the velar muscular sling (14, 15). Numerous studies applied these two techniques to cleft repair and evaluated the postoperative outcomes respectively. However, limited studies performed a definitive comparison of velopharyngeal morphology between them. Herein, the comparisons were made between the two techniques in several methods, revealing that better speech outcome was produced in Furlow palatoplasty as opposed to Sommerlad palatoplasty.

VPC is the premise to yield adequate speech intelligibility. It was reported that 5% to 30% of patients have unsatisfactory speech outcomes and require secondary treatment because of velopharyngeal insufficiency (VPI) after primary palatoplasty (23–34), which may be related to several risk factors, including short velar length, insufficient velopharyngeal motility and deep palatopharyngeal cavity (26). Consistent with previous studies, our data suggested that the VPC rate was 90% in Furlow palatoplasty group vs. 81.3% in Sommerlad palatoplasty group. Furthermore, poorer speech intelligibility, higher hypernasality and nasal emissions were displayed in our study. This difference between these two methods is possibly due to the surgical characteristics that exert influence on the outcome of the operation. Both the Sommerlad and Furlow techniques repositioned the displaced levator veli palatini, contributing to develop a functional velum. Sommerlad palatoplasty focused more on anatomical reconstruction of the abnormal levater veli palatine and enhancement of muscular function, while the extension of the soft palate length is limited, which influences the outcome of palatopharynx closure. Nevertheless, Furlow palatoplasty not only restored the velar muscular sling to proper orientation but also elongated the velar length reducing the risk of longitudinal scar contracture. Consequently, the Furlow repair has a stronger ability to extend the soft palate length than sommerlad palatoplasty (18). After 289 patients with cleft palate repaired by Furlow palatoplasty, Chorney et al. (27) found only 5% of the patients needed secondary surgery as a result of VPI. And Wang et al. reported the length of soft palate increased by 13.23%, 10.10% after Furlow and Sommerlad palatoplasty, respectively (18). In line with these results, lateral cephalogram evaluation revealed a significantly increased soft palate length and higher adequate ratio after Furlow palatoplasty compared with Sommerlad palatoplasty, while there were no significantly differences in velopharyngeal depth between them.

Vlopharyngeal function assessment in previous studies depended largely on perceptual examination (8, 28, 29), which existed with the defect of significant heterogeneity and prevented comparison of results from different centers. Several methods have been developed to assess and describe velopharyngeal function and speech outcome, including electromyogram and nasopharyngeal endoscopy, but none of the existing methods can assess all indexes independently (30–34). To ensure a more reliable and accurate evaluation of velopharyngeal function and speech results in cleft patients, various evaluation methods were applied simultaneously in our study. Apart from perceptual assessment, 2-dimensional cephalometric studies provide a static evaluation of the velopharynx, while nasopharyngeal endoscopy can record and immediately replay velopharyngeal closure. Nasoendoscopy played a critical role in determining VPI, especially marginal VPI, which perceptual speech evaluation was in doubt. Therefore, jointly applying these methods can provide compelling data that can be made through comparison and analysis between different centers.

Of course, this present study exists certain limitations that should be acknowledged.

A notable shortcoming of our research is the insufficient sample size. Further data collection is required. Besides the surgical technique, for the lack of standardization of variables such as cleft width and hard palate repair technique, further analyses based on the above variables will be conducted in future research. The present study revealed that Sommerlad technique yielded worse velopharyngeal function and speech outcomes compared to Furlow technique. For HSCP patients, relative to Sommerlad palatoplasty, Furlow palatoplasty may be a more suitable surgical option. Of note, the opposing Z-plasty sacrificed velar width to gain length, which is not applicable to all types of clefts, particularly wide clefts due to excessive tension.

## Conclusion

Furlow technique was proven to be superior compared to Sommerlad palatoplasty in terms of velopharyngeal function and morphology following primary cleft palate repair, which showed better speech intelligibility, higher VPC rate, longer velar length, shorter pharyngeal depth and a bigger Adequate ratio. Based on the above results, the Furlow palatoplasty should be preferred for patients with HSCP.

## Data Availability

The raw data supporting the conclusions of this article will be made available by the authors, without undue reservation.
